# Computer-based cognitive rehabilitation program GRADIOR for mild dementia and mild cognitive impairment: new features

**DOI:** 10.1186/s12911-020-01293-w

**Published:** 2020-10-22

**Authors:** Manuel A. Franco-Martín, Angie A. Diaz-Baquero, Yolanda Bueno-Aguado, María T. Cid-Bartolomé, Esther Parra Vidales, María V. Perea Bartolomé, Isabel de la Torre Díez, Henriëtte G. van der Roest

**Affiliations:** 1 Psychiatric Department, Rio Hortega University Hospital, Valladolid, Spain; 2 Zamora Hospital, Zamora, Spain; 3 Institute of Biomedical Research of Salamanca, University of Salamanca, Salamanca, Spain; 4 Department of Research and Development, Iberian Research Psycho-Sciences Institute, INTRAS Foundation, Zamora, Spain; 5 GRADIOR Department and Cognitive Research, INTRAS Foundation, Valladolid, Spain; 6 Technology Development, INTRAS Foundation, Valladolid, Spain; 7 IBIP Center for Clinical Care in Mental Health and Aging, INTRAS Foundation, Zamora, Spain; 8 Basic Psychology, Psychobiology and Methodology Department, Salamanca University, Salamanca, Spain; 9 Department of Signal Theory and Communications, University of Valladolid, Valladolid, Spain; 10 Department on Aging, Netherlands Institute of Mental Health and Addiction (Trimbos-Institute), Utrecht, The Netherlands

**Keywords:** Dementia, Software, Neurological rehabilitation, Cognition, Community mental health services

## Abstract

**Background:**

The growing number of older people and, with it, the increase of neurological impairments such as dementia has led to the implementation of the use of computer programs for cognitive rehabilitation in people with dementia. For 20 years, we have been developing the GRADIOR cognitive rehabilitation program and conducted several studies associated with its usability and effectiveness. This paper describes the development of the latest version of the GRADIOR computer-based cognitive rehabilitation program for people with different neurological etiologies, especially mild cognitive impairment and mild dementia.

**Results:**

GRADIOR is a program that allows cognitive evaluation and rehabilitation of people affected by cognitive impairment. The new version of GRADIOR is characterized by a structure that is dynamic and flexible for both user and therapist, consisting of: Clinical Manager, Clinical History Manager, Treatment Manager and Report Manager. As a structure based on specific requirements, GRADIOR includes a series of modalities and sub-modalities, each modality comprising a series of exercises with different difficulty levels.

**Discussion:**

Previous studies associated with earlier versions of GRADIOR have allowed the development of a new version of GRADIOR. Taking into account aspects associated with user experience, usability and effectiveness. Aspects that have made it possible to achieve a program that can meet the needs of older people with dementia.

## Background

Europe is an ageing society. Eurostat’s population projections anticipate that in the coming decades the number of people aged over 60 will increase by approximately two million people per year, accounting for around 30% of the total population by 2060 [1]. Dementia and cognitive impairment are age-related conditions that involve very high healthcare demands. The overall crude prevalence rate for mild cognitive impairment (MCI) in the over-60 population is between 6 and 42% [2], and with 20–40% of such cases progressing into dementia [3]. Approximately 5–7% of the world population has developed some form of dementia [4]. In Spain alone, over 800,000 people are affected by dementia [5].

Due to its high prevalence and consequences in the older population, dementia has become a major public health challenge [6] and a healthcare priority in many countries [4]. Projections based on current healthcare policies predict an increase in age-related public expenditure from 4.1% to around 29% of Gross Domestic Product by 2060 [7]. Such rising costs will put a strain on the sustainability of existing healthcare systems [8]. To counteract the rising health care expenditures, European policies are increasingly focussing on independent living for older adults, since community care is cheaper than care in a facility.

In recent decades, many different psychosocial approaches aimed at improving and maintaining cognitive ability have been developed to slow down the progression of dementia as much as possible and to enable people affected by it to age healthily [9, 10]. Various studies have proven the positive effects of cognitive rehabilitation as an individualised cognitive intervention explicitly focused on a person's objectives and needs (cognitive profile) [11]. Huckans, Hutson [12] reported improvements in performance in people with MCI in at least one cognitive domain, which shows that adults with MCI are still able to learn. Another study found that cognitive rehabilitation had a long-lasting effect on the overall cognition of older adults experiencing age-related cognitive decline [13]. Recent studies suggest that slowing the progression of dementia by one year would lead to a better quality of life for its sufferers [14, 15] and to a significant cut in the related socioeconomic costs [16–18].

The most common implementation of cognitive rehabilitation is based on pen and paper exercises and training that is conducted by a neuropsychologist. This makes the treatment very costly, which added to the fact that it is not easy to have a neuropsychologist available in every treatment location (e.g. community or primary care center), means that accessibility to this approach can be poor. It is well known that people with dementia in Europe have trouble in getting access to adequate treatment, especially to psychosocial therapies [19]. Particularly in rural and semirural regions of the vast majority of European Union countries, where the percentage of people over 65 years of age is above national averages and resources for services or treatment are scarce [20, 21].

Since timely treatment is crucial to achieve better results and fewer complications, it is important to improve accessibility to services and treatments [21]. A good opportunity to increase accessibility to treatments could lie in Information and Communications Technology (ICT) solutions for health and wellness coaching. There are already studies that have shown that computer-based cognitive interventions are effective in improving cognition, anxiety and mood in people with dementia, and can lead to better results than non-computer-based interventions [22, 23]. Nevertheless, despite technological progress, the improved user-friendliness of ICT devices and the spread of smart phones, tablets and other wearables, the use of new ICT solutions for people with dementia is still very low.

**Fig. 1:**
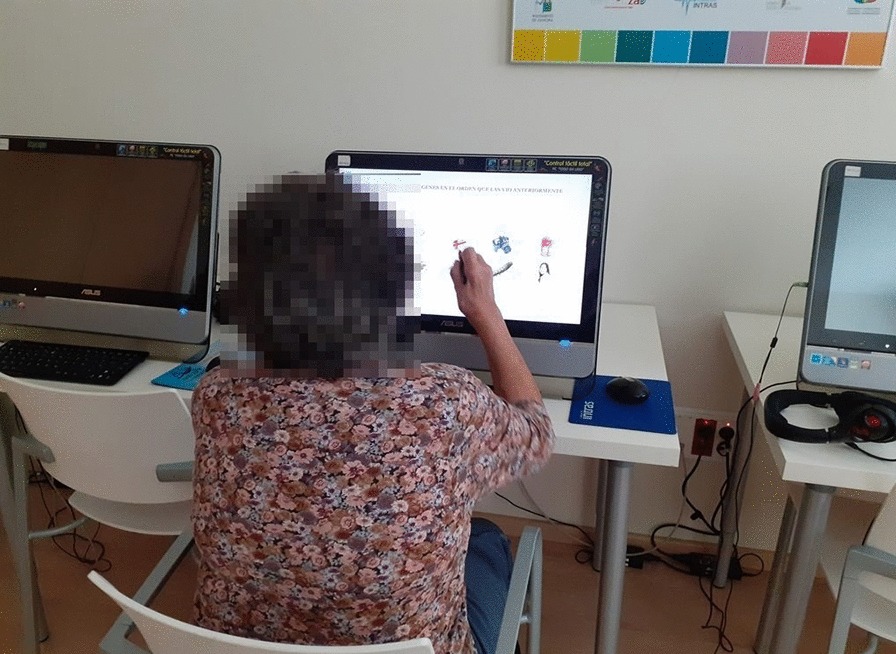
Person with dementia performing cognitive stimulation with software GRADIOR

Cognitive computer-based training programs still face the challenge of being accepted by elderly people who are not very familiar with technology [24]. In addition, these solutions must be embedded into the strategies and goals of the end-user organisations, service providers and business partners, which requires these tools to be user-friendly and useful for therapists, and well-accepted by carers and patients. From the INTRAS foundation, we have tried to improve care for people with cognitive impairment by developing a new computer-based tool for cognitive rehabilitation called GRADIOR (Fig. 1). In constant development for the last 20 years, the earliest version of GRADIOR has been used in clinical practice since 1997, adding improvements ever since. This paper describes the development of the latest version of the GRADIOR computer-based cognitive rehabilitation program for people with different neurological etiologies, especially mild cognitive impairment and mild dementia.

GRADIOR is a computer-based program used for neuropsychological rehabilitation in people suffering from one or more cognitive disorders of different etiology, as well as for cognitive stimulation in healthy people [25]. GRADIOR was designed to stimulate the full range of cognitive skills and also includes tools for neuropsychological assessment. The program uses a touch screen in order to make its use easier for people lacking computer literacy. The development of GRADIOR started 20 years ago, ever since combining knowledge on neuropsychological advances in the field of clinical expertise with the experiences of end-users and stakeholders in its development process.

The first GRADIOR version was funded and validated by the Social Affairs Minister [26]. The third GRADIOR version was acknowledged by the 2007–2010 Alzheimer Plan of Andalucía (Spanish region) as recommended software for cognitive stimulation. Currently, there are more than 500 clinical and social settings in Spanish cities that use and support different GRADIOR versions as a good rehabilitation and stimulation cognitive tool. GRADIOR is used by more than 11,000 people.

### Requirements

#### Non-functional

In this regard, the new GRADIOR version was designed pursuing the following objectives: (a) a familiar looking interface, (b) to facilitate the role of professionals through the creation of new exercises, making it possible to obtain user-performance reports and digitized evaluations, (c) an easy-to-use program based on a tactile interface, (d) a program with exercises that allow the improvement or maintenance of cognitive functions, (e) as well as, a program to promote the socialization of older adults with other people who have the same needs and/or problems.

GRADIOR is based on a series of essential features that make it an easy access program that offers adequate user experience. Thus, it is (a) flexible: it has been developed for a broad variety of disorders such as: neurodegenerative diseases, brain damage, stroke, mental retardation, mental illnesses, and epilepsy. Therapists can tailor the rehabilitation approach to the patient’s cognitive profile, personal preferences, and needs; (b) dynamical: it allows the addition of new tools; (c) user-friendly: it can be used by users who lack computer-literacy; (d) economical: it is easily accessible and accommodates the economic needs of its target population; (e) highly accessible: it can be easily implemented in any setting, including rural areas; (f) useful: studies on earlier versions reveal positive results for this program [26].

#### Functional

Likewise, certain functional requirements were established for the program, which should enable therapists to: (a) obtain a neuropsychological profile of each user based on the neuropsychological evaluation of each of the cognitive processes included: orientation, attention, memory, language, reasoning, calculus, executive function; (b) design and implement an individualized cognitive training plan according to the cognitive processes affected and the level of deterioration in each of them; (c) periodically adjust the treatment plan to the patient's performance and continuous improvement; (d) draw up user performance reports based on the exercises, sessions, modalities and sub-modalities, thus facilitating work on adjusting the plan and producing progress reports.

#### Technical

GRADIOR software is compatible with a Windows operating system (the current version of GRADIOR requires Windows 7 SP1 or later) and is specifically designed for touchscreen computers, although it can be also used with the mouse or keypad. To run smoothly, the system requirements are: RAM (2 GB/4 GB recommended), graphics card (RAM) (256 MB minimum/1 GB recommended for optimal graphical performance) and Microsoft Office 2003 or later versions. We are currently working on a tablet version.

Minimum requirements for the PC where the server is to be installed:

- Windows server 2008, 2 GB RAM + 3.2 GHz + .NET Framework 3.5 SP1.
- Configuration of antivirus exclusions to allow remote access.
- Wired internet connection.
- IP publishes FIXED and configuration of the necessary ports to access the SQLSERVER from an external client (if the server is not in the same NETWORK).
- Local administrator permissions.

Minimum customer equipment requirements:

- Operating System: Windows 7 ServiPack1 + .NET Framework 3.5 SP1.
- RAM: 2 GB minimum—4 GB recommended.
- Graphical performance: 3.0 MHz minimum—4.5 MHz recommended.
- RAM graphics card: 256 MB minimum—1 GB recommended.
- Tools installed: Microsoft Office 2003 or higher.
- Configuration of antivirus exclusions to allow the running of the GRADIOR program.
- Wired internet connection.
- Local administrator permissions.

### Implementation

Microsoft Visual Studio is used as an integrated development framework for GRADIOR. The object-oriented programming language that brings together all the necessary components for developing applications is Visual Basic.NET. SQL Server acts as the database management system.

Easy development methodology: Scrum is an agile, incremental and iterative development framework. It allows the planning and managing of the development, focusing on achieving high productivity and quality levels while mitigating the risks of software development thanks to a regular review and adjustment of the process and product.

Some of the benefits for which Scrum has been selected are:

- Project status and progress visibility.
- Systematic mitigation of risks by means of intensive phases. The complexity of the development is reduced (requirements, technology) to what fits in one sprint.
- Enhancement of product internal quality to be built incrementally and at a constant pace.

### Architecture and deployment

GRADIOR 4 has been developed according to a “client/server” architecture that allows users to execute the “client” on their own device, which connects to a server that stores the data shared by all the “clients”. This architecture is supported by the Microsoft Framework.NET platform. In this way, GRADIOR can be easily implemented in home care.

In short, GRADIOR 4 supports three installation options:

- Basic installation (single-user system): For local use, both “server” and the “client” are installed on the same device (internet connection not required).
- Installation in local area network (intranet): GRADIOR is installed according to a standard “client/server” architecture. Data are stored on a local server (internet connection not required).
- Remote mode installation: GRADIOR is installed according to a multi-site architecture. The “server” (database management system) is installed on the main premises while “clients” can be deployed in far-away facilities (internet connection required) or in main facilities (intranet access or internet access).

Through the server, the therapist has access to the data of every treatment session, while also acting as an administrator who can modify and personalize treatments for every user from the server computer. Likewise, the “server” computer allows the therapist to video contact the client. GRADIOR is available in Spanish and English, but its contents can be easily changed and culturally adapted to any language or environment.

## Results

This section describe different structural and functional aspects of the GRADIOR rehabilitation program: the modules that comprise it, the different steps to plan an intervention plan and preliminary data on usability from previous versions of GRADIOR. Finally, this section places special emphasis on aspects of usability and user experience of the new GRADIOR 4 version, citing studies that support it.

### Description of the program

GRADIOR has been developed to design and manage personalized cognitive rehabilitation treatments, save patient clinical features, overview results and adapt exercise difficulty to the patient’s cognitive level. The current version includes eight different moduli (orientation, memory, attention, calculus, executive function, perception, language and reasoning) for clients to follow.

Therapists have the following five GRADIOR functionalities at their disposal:

#### Clinical management

**Fig. 2:**
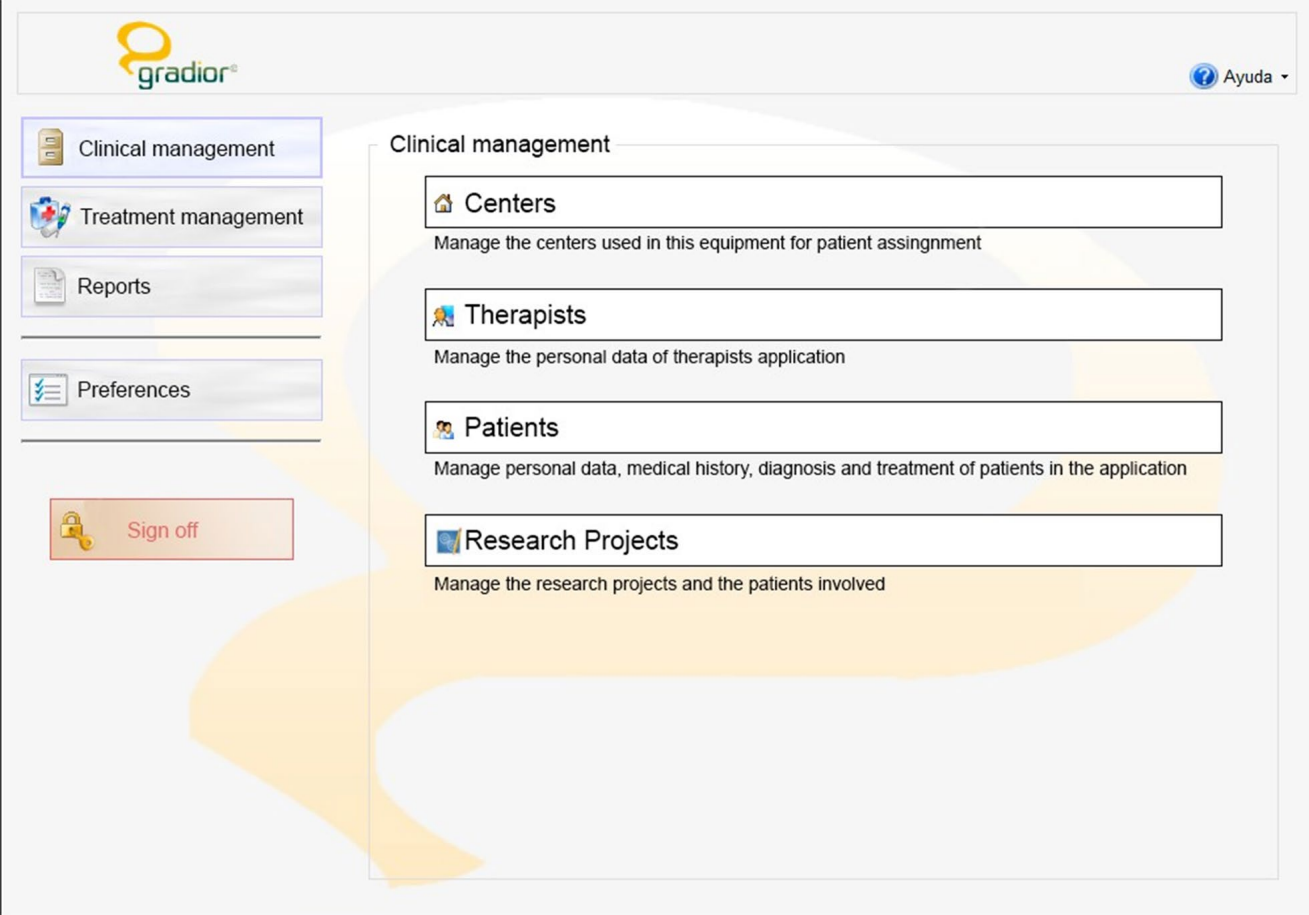
Clinical management in software GRADIOR

**Fig. 3:**
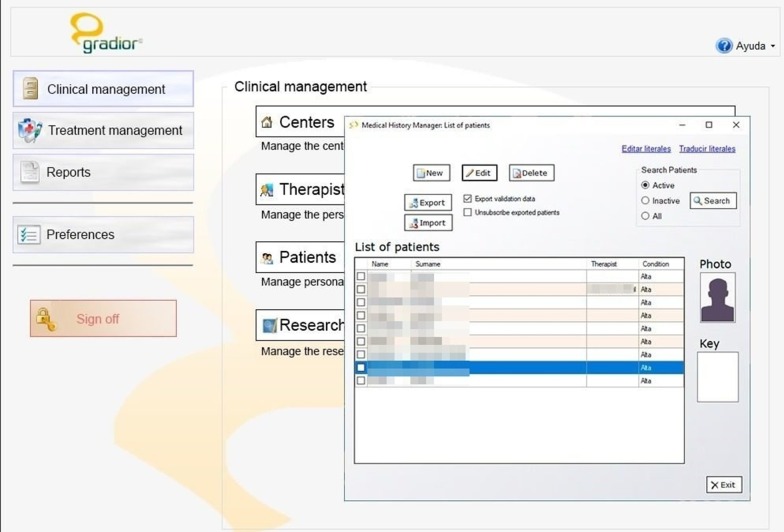
Medical history manager: list of patients in software GRADIOR

The Clinical Management provides an overview of the user accounts of all the patients under treatment by a specific therapist, also allowing the addition of new users to GRADIOR (a picture and password are required for every patient), the modification of information and the deletion of user accounts. The therapist can see the centre, appointed therapists and condition of every patient (Figs. 2, 3).

#### Clinical history manager

The Clinical History Manager stores the (socio) demographic and clinical data of every GRADIOR user. Client files such as personal data, clinical observation, medication and results of clinical assessments are stored in a session that can be resumed. The therapist is the only person authorized to access this session through two-step authentication. In a clinical observation session, the therapist can record the diagnosis from the International Classification of Diseases 10 (ICD-10), record the patient's illnesses and family background. In the medication session, the therapist can register information about medication, e.g. dose, duration, start date and end date.

**Fig. 4:**
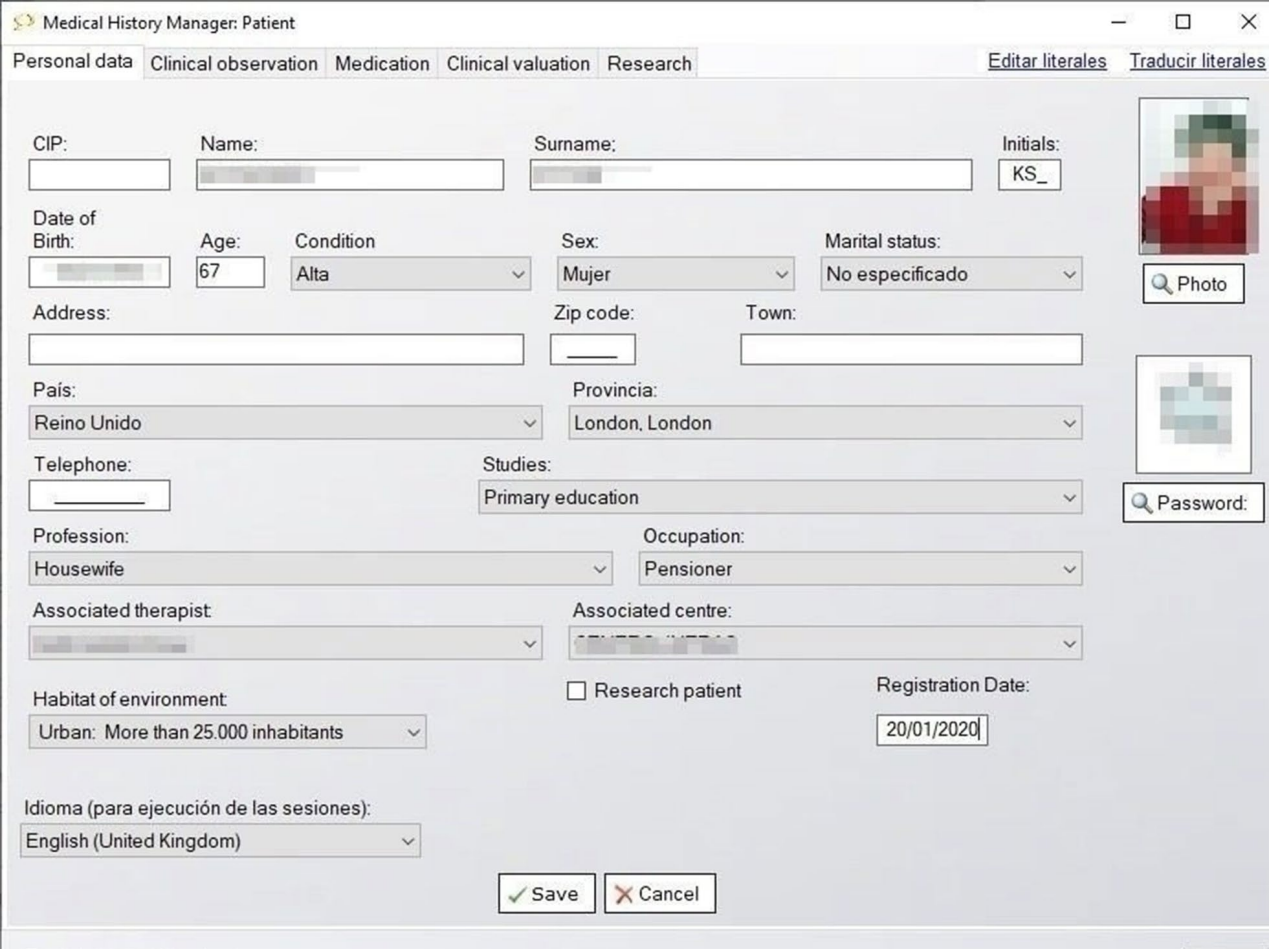
Medical history manager: patients in software GRADIOR

In results of clinical assessment, the scales used in people with cognitive impairment are, in this order: Mini Mental State Examination (MMSE) [27], Barthel Scale [28], Geriatric Depression Scale Yesavage (GDS) [29], Lawton Instrumental Activities of Daily Living questionnaire (IADL) [30], Cambridge Cognition Examination (CAMCOG) [31], Clock Drawing Test (CDT) [32] and Trail Making Test (TMT) [33] (Fig. 4).

#### Treatment management

The Treatment Management is a key function. Here the therapist can design personalized treatment plans based on the information of the user’s cognitive profile as stored in the Clinical History Management, unmet needs and preferences. The therapist can schedule trials, select exercises by cognitive sub-modalities, establish levels of difficulty for each exercise and define the duration of the individualized cognitive rehabilitation plan.

**Fig. 5:**
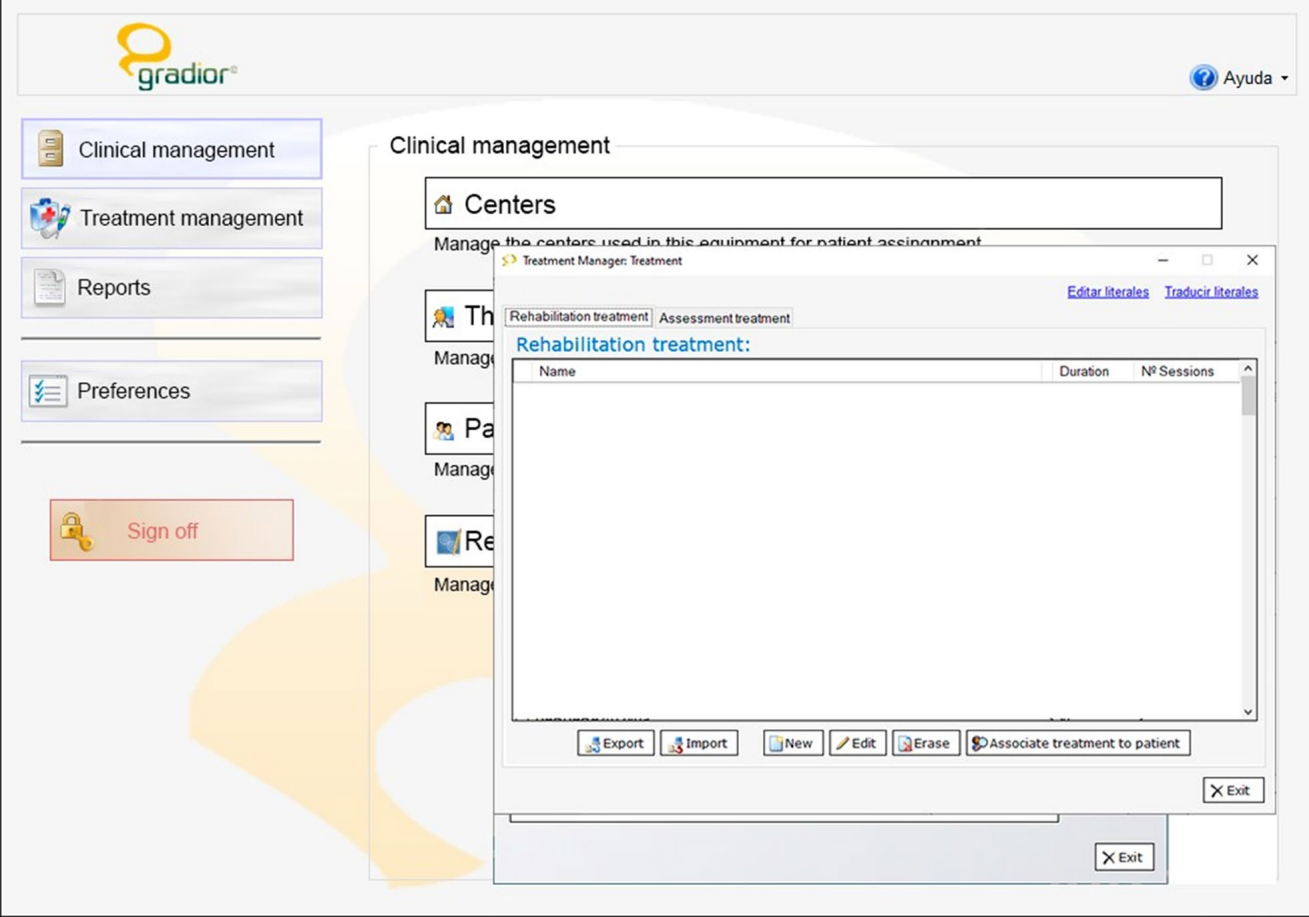
Treatment management in software GRADIOR

In most cases, the therapist designs a one-week trial rehabilitation training plan, which is subsequently fine-tuned to a suitable personalized cognitive rehabilitation treatment according to the patient’s performance. Training can be adjusted at any time during the course of the program, depending on the follow-up and performance of the patient. The program contemplates no automatic changes in treatment plan, so that any alteration always requires the intervention of a therapist (Fig. 5).

#### Report manager

**Fig. 6:**
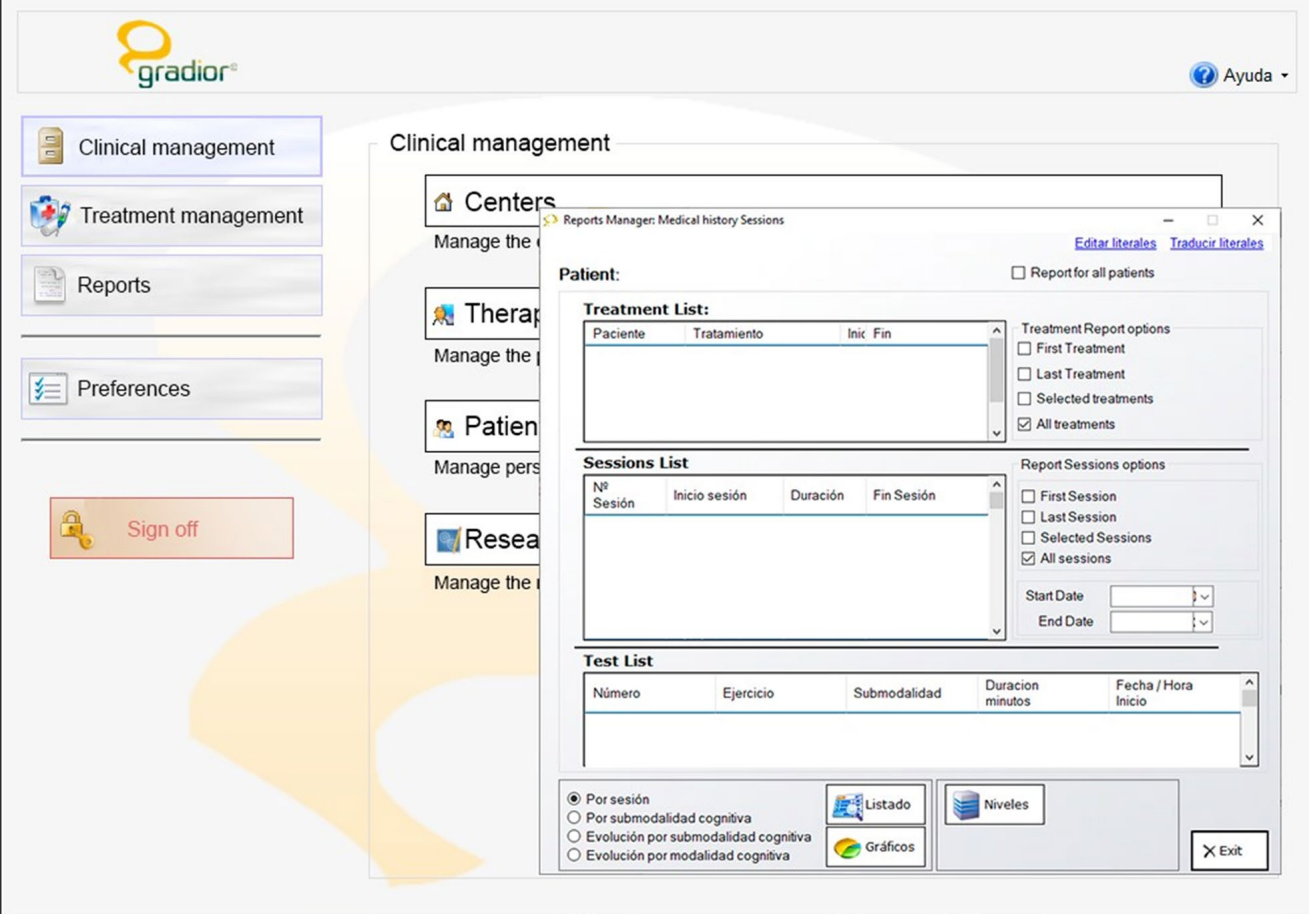
Report manager: medical history sessions in software GRADIOR

The Report Manager stores the performance data obtained from every user trial separately. It allows the tracking of patient improvement over time for all cognitive functions. This is essential for patient monitoring and to adapt the cognitive rehabilitation intervention to the user’s needs. The therapist can obtain different types of patient report, e.g. report by modality and sub-modality at a general level or level-specific reports. This last report helps the therapist to modify the levels according to individual patient performance (Fig. 6).

#### Modalities, sub-modalities and exercise/task description

**Fig. 7:**
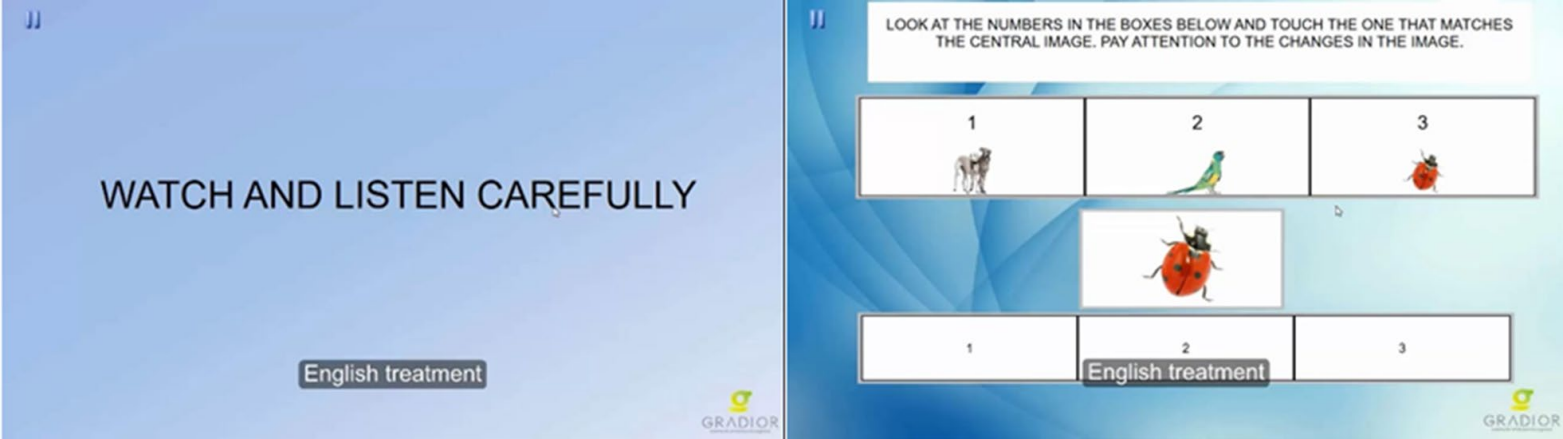
Attention exercise in software GRADIOR

GRADIOR includes exercises aimed at stimulating a variety of cognitive functions (modality): orientation, attention, calculus, executive function, language, memory, perception, reasoning, and different sub-modalities of every function (Fig. 7). Every sub-modality includes different performance levels and different types of exercises (Table 1). Some examples can be found on the GRADIOR website https://www.intras.es/nos-hacemos-mayores (See additional file 1). For instance, the different sub-modalities of the memory modality are: long-term graphic memory, immediate verbal memory, short-term verbal memory, short-term verbal memory compound, long-term verbal memory, implicit memory, location memory, semantic memory and span memory direct.

**Table 1:** Cognitive modalities and sub-modalities addressed by GRADIOR 4

Cognitive modalities	Cognitive sub-modalities (levels)	Cognitive modalities	Cognitive sub-modalities (levels)
Orientation	Temporary orientation [3]	Memory	Associative memory face-name [6], associative memory image-word [6], associative memory word-word [6], auditory memory (like verbal memory), immediate graphic memory [7], short-term graphic memory [7]), short-term graphic memory compound [7], long-term graphic memory [7], immediate verbal memory [7], short-term verbal memory [7], short-term verbal memory compound [7], long-term verbal memory [7]), implicit memory [1], location memory [6], semantic memory [1], span memory direct [8], span memory reverse letters [8], span memory direct numbers [8], span memory reverse numbers [8], direct object span memory [8], span reverse object [8]
Attention	Sequential visual selective attention [7], simultaneous visual selective attention [3], attention vigilance color [7], attention vigilance flashes [7], sustained attention color [2], sustained attention sparkles [2], sustained attention figures [2]		
Calculus	Quantitative calculation counting [5], calculus identification of numbers [3], calculus arithmetic problems [12]		
Executive function	Change rules [2], key task [6], auditory inhibition [4], visual inhibition [9], interference [7], numbers and letters [7], ordination stories [4], puzzles [10]	Perception	Perception colors auditory [11], perception colors graph [11], perception colors text [11], visual perception figures [4], visual perception faces [3], visual perception sizes [3]
Language	Language comprehension of words [2], language identification of written letters [3], oral letters identification language [3], word recognition language [2]	Reasoning	Reasoning sorting graphics [2], reasoning sorting texts [2]

A special *module called *“INTRAS” permits access to the GRADIOR content database that contains the material used for the exercises (pictures, words, voices, recordings etc.) and allows the addition of new content. The therapist can add new or specific content to the database that meets the preferences of the patient, takes into account the cultural environment or follows a stimuli ontology. Such new content can even be of personal significance to the individual patient, including family pictures or familiar voices. The module enables the description and categorization of all the stimuli that are required to develop new exercises or to automatically translate the system into other languages.

The INTRAS module is key to the high flexibility and personalized training plans provided by GRADIOR. Therefore this is essential to maximize the benefits patients can obtain from the cognitive rehabilitation program. This module is where the difficulty level for each cognitive sub-modality can be defined, according to the complexity of the stimulus, the number of stimuli, the speed of presentation, the perceptual modalities, familiarity with the stimuli or the number of confusion stimuli. Although GRADIOR’s open functioning and flexibility involve many advantages, these could also be a barrier.

Due to the large number of possibilities, defining a therapeutic plan can be very time consuming for therapists. To avoid this, the INTRAS module is optional and therapists can use all the exercises previously developed by default in the GRADIOR 4-basic. The program contains 12,601 exercises (attention-1533, perception-1104, memory-4674, calculus-1500, language-452, reasoning-404, and executive function-2934). This means that the variability of exercise and task is wide enough to avoid repetition during a cognitive rehabilitation program.

Users personalize their treatment by selecting a personal identifier (photo/name) and the traditional password is replaced by a picture chosen by the patient. At the beginning of every session, patients select their personal image and then their password out of 9 pictures by touching the screen. Thus, many users can follow their personalized cognitive rehabilitation program on the same computer without the need to remember a password.

How does it work? GRADIOR-based cognitive rehabilitation treatment steps.

GRADIOR-based cognitive rehabilitation follows a five-step protocol that is implemented in the following order:

1. Preliminary neuropsychological assessment and baseline definition.
  Before starting cognitive rehabilitation, it is necessary to obtain the user’s cognitive profile in order to personalize the approach. Therefore, the first step is to apply a traditional comprehensive neuropsychological assessment by means of a test battery in GRADIOR.
2. Trial using baseline treatment.
  GRADIOR offers standard treatments for people with similar cognitive capacity. Based on the cognitive assessment results, the user will receive a standard one-week training schedule. This trial week allows the therapist to acquire a thorough insight of the user’s cognitive skills, computer skills and motivation to use GRADIOR.
3. Designing personalized cognitive rehabilitation.
  The trial provides the therapist with the appropriate knowledge of the patient’s skills and preferences to design a personalized treatment plan. The therapist chooses the exercises to be included in the cognitive rehabilitation treatment plan, their difficulty level, establishes session frequency and duration. The therapist controls all the cognitive rehabilitation variables.
4. Providing personalized cognitive rehabilitation.
  After these three steps, the user starts the actual personalized intervention. Training sessions are completed at home or at a convenient location (e.g. hospital, community center) according to a pre-fixed schedule. While performing the exercises, users can receive feedback on their scores and skills at the end of every session. Although this is optional, it might increase motivation to follow the sessions.
5. Fitting treatment regularity (levels of difficulty, frequency of tasks).

Depending on the user, the plan can be adapted every month. The therapist checks the patient’s outcomes over the last period and makes the necessary changes in order to adapt the cognitive rehabilitation plan according to new unmet needs or improved cognitive skills. The client’s feelings and motivation regarding GRADIOR are discussed. In the case of patients living in faraway locations, this is discussed via a videoconference embedded in GRADIOR.

### GRADIOR usability and usefulness. Preliminary data

From its earliest versions, the GRADIOR program has reached high levels of user satisfaction and usability, contributing to the alleviation of neurocognitive symptoms in people with different pathologies [34]. GRADIOR also provides support for therapists in their daily work, since they report satisfaction with its usefulness and consider it a good psychostimulation tool [35].

The usability characteristics: easy learning, effectiveness/efficiency, memory capacity, low error rate and satisfaction were measured through a "satisfaction evaluation of the GRADIOR centers". It was conducted by Zoto, leader of a Technology Research Group at the Polytechnic University of Madrid. In terms of usability, GRADIOR was considered [34]:

- Highly acceptable, due to its flexibility and simplicity;
- Highly user-friendly, due to its welcoming and approachable interface;
- Highly satisfactory for therapists and users in terms of contents.

Therapists pointed out that it was necessary to continue developing content on cognitive modalities, especially on execution skills. And even though GRADIOR was initially created for people with no technological skills; already in the first version, therapists sometimes felt that certain technical capabilities were required to use the program [34].

Despite these findings, the overall data proved GRADIOR to be highly acceptable. Even among people with schizophrenia, 83.1% of its schizophrenic users enjoyed working with GRADIOR [35], whereas only 22.9% of them reported difficulties in using the program. Most of the respondents considered that GRADIOR had a welcoming interface and that it was pleasant to use.

Not only aspects of usability were defined, aspects associated with effectiveness were also evaluated. GRADIOR proved effective in treating behavioral and cognitive symptoms. In total, 61.8% of patients reported an improvement in their quality of life and independence, and 77.1% of the people described GRADIOR as a useful tool for the provision of individualized treatment according to their needs [35]. Also, other studies found that GRADIOR allowed maintenance of cognitive functions and improvement of emotional and behavioral aspects in people with mild dementia and MCI [36].

### Latest development: GRADIOR 4

Drawing from the results of the first studies, new features were proposed in order to improve GRADIOR’s efficiency and usefulness. Suggested improvements included: development of a telematics network for easy understanding of instructions, variety of exercises and levels of difficulty, use of good color contrast aided visibility, changes in software programming to avoid interruptions [34] and the inclusion of real images in the exercises would help them to be more familiar and real for the patient [37]. All these features were introduced in the latest version, GRADIOR 4.

The development of GRADIOR was oriented in a user-centered design. In this way, GRADIOR responded to the needs and characteristics of the target population, generating greater usability [38, 39].

Considering the above, subsequent usability studies were carried out with the latest version of GRADIOR. The first studies reported that 81.2% of patients generated an acceptance of the program [40]. Moreover, 91.1% of the patients reported that they enjoyed the sessions, 63.3% of the patients mentioned that the instructions were clear and understandable and 70% of the patients reported that the program met their expectations [41].

Toribio Guzmán [42] proposed a study on aspects of usability and user experience of version 4 of GRADIOR combined with a physical program (Long Lasting Memories Program). For this objective, this study took into account different steps: (1) screening the population by applying the MMSE and the GDS, (2) phase of adaptation and learning to the program, (3) intervention for 3 months: 3–5 days a week for 40 min of cognitive training and 3 times a week for 1 h of physical training, (4) supervision during the sessions, (5) usability evaluation through the design and use of a questionnaire consisting of 5 dimensions: affective evaluation, usability, satisfaction, sustainability, independent life and social integration.

The results of the previous study are presented below. In the dimension on affective evaluation, the patients generated positive feelings and reactions to the use of this program, 79% of patients expressed that the program was fun and 78.9% of patients did not show feelings of boredom [43].

In the usability dimension, a good usability was highlighted through values that exceeded 87% in each evaluated usability criterion (attractive design, images, features in the physical-mental exercises, the main menu and exercises adapted to physical and mental abilities) [44]. A total of 60.1% of the people with MCI established that it was easy to use. In contrast, 40.1% of the people with mild dementia expressed difficulty in its use, requiring help or support during training sessions [43].

In the dimension of satisfaction, a clear predisposition to use the program was highlighted [44]. A quantity of 83.7% of patients believed that it was beneficial to their health. A total of 73% of the participants indicated that the program met expectations. And in 66.9% of the patients, there were feelings of security regarding the use of a technological device [43].

In the dimension of sustainability, 84% of the patients expressed interest in continuing to use the program [44], 96.1% of the patients would recommend the program and 78.1% of the participants would pay for the program [43].

In the last dimension, the patients noted the increase in their social interaction [43]. A total of 37.4% of patients thought they could use it independently at home [44] and a representative score in independent and social life was highlighted for the group with MCI compared to healthy participants.

However, an analysis of the usability of the alpha-version of GRADIOR 4 revealed that, while 46.5% of its users could work easily, it depended on age and on the severity of the impairment suffered [37].Therefore, the user-centered design of any program associated with cognitive training should take into account the characteristics of cognitive decline (type, level, and deficits) of people with dementia.

In this way, the program will be more usable and more widely adopted to the target population [39] As mentioned above, GRADIOR has different exercises per sub-modality for each modality and at the same time, these exercises have different levels of difficulty, which allows the program to be adapted to the type and level of cognitive decline of the patients.

Additionally, physical disability was found to seriously limit access to GRADIOR. In other words, people with impaired mobility may have difficulties moving to centers where the program is taught. Hence, the GRADIOR version for tablet has been developed; in this way, people can access the program from their own home without having to move to a center.

While context should be taken into account in the design of any technology, not all technologies are applicable to rural environments [38]. Nevertheless, the latest GRADIOR version is applicable in different environments and accessible to people living in rural areas [45]. Indeed, the possibility of using GRADIOR at home through remote monitoring was defined as a priority for future developments [42].

Finally, different professionals who had used version 4 of GRADIOR combined with a physical program mentioned the following: 100% of professionals thought that patients enjoyed the sessions, 60% of them indicated GRADIOR as an easy-to-use program and 100% of professionals rated GRADIOR as a beneficial program [43].

## Discussion

After 20 years of development, GRADIOR has become an easy to use and implement computer-based cognitive rehabilitation program, particularly in clinical settings.

Over the last decade, several computer-based cognitive rehabilitation programs have been developed, targeting people at risk for cognitive decline [46, 47]. Many of them have shown positive effects on cognition in different user groups, for instance in preventing cognitive decline in healthy older adults [48] and people with Alzheimer’s disease [49]. These programs improve cognitive skills or delay impairment caused by MCI or dementia [50–53].

The common benefits of these computer-based training programs as compared to ‘traditional’ cognitive rehabilitation programs are the high accessibility of the treatment and its flexibility to adapt the training according to the user’s needs, cognitive capacities and motivation [54, 55]. Additional aims of these programs can be to stimulate some of the limited or impaired physical skills of their users (e.g. grasping, arm movement, etc.) [56] or to relieve caregivers’ burden [57].

The main advantage of GRADIOR as compared to other computer-based cognitive rehabilitation programs is its flexibility, which allows complete personalization of the training according to users cognitive skills, needs and familiarity with the content [39]. Exercises are based on real pictures, drawings, 3D-virtual objects, sounds, voices, videos and a wealth of multimedia resources aimed at maintaining a high level of attention throughout the sessions. Because of their large number, exercises can be randomly applied avoiding repetition in a same training program and, thus, reducing user boredom. The additional INTRAS module allows therapists to design more exercises.

In general, technological applications are well-accepted by young people, but older or disabled people (target users) might find difficulties or barriers in their use, which makes them reluctant to adopt new technology. To optimize the use of GRADIOR, we used the interface of existing mainstream technology: a touch screen. When touchscreen devices appeared, the computer–human interaction became more intuitive [58] and such devices can be used easily without prior experience, even by people with dementia or MCI [59, 60]. To avoid rejection of the GRADIOR interface, the computer was converted into a TV, an object that is very familiar to elderly people. Not needing to use keyboards contributes to the high usability of GRADIOR. To support this arrangement, usability studies were conducted with old people and schizophrenic patients [61].

Patients identified GRADIOR as an easy-to-use program [43] and generated an acceptance level, especially in people who have never used a computer [40]. GRADIOR noted for its high level of usability by meeting the parameters associated with this construct [44]. Designated as a program that generated positive health benefits [43] and met patient expectations [41]. GRADIOR not only generated the maintenance of cognitive functions, but also increased in social interactions and mood [43]. These contributed to increase predisposition in patients to continue using GRADIOR [43, 44], due to the positive experience of enjoyment in its use by the user/patient.

GRADIOR has also proved to be cost-effective as the Report Manager Module helps therapists save a lot of time when analyzing patient performance data. The monitoring of clients is very easy and the outcomes can be used as feedback for patients.

In general, GRADIOR provides most of the advantages of using technologies for cognitive rehabilitation. Recently, Zokaei, MacKellar [62] proposed some recommendations for computer-based cognitive training programs in order to increase their success. Their recommendations are that the program (a) targets specific cognitive functions (e.g. memory, attention, etc.); (b) this can be continuously adapted based on participant performance; (c) this will be very immersive and entertaining; (d) this includes immediate quantitative feedback; (e) this is highly accessible from portable digital devices.

GRADIOR meets all of the above: (a) GRADIOR allows stimulation and training for each of the cognitive functions: memory, attention, language; (b) GRADIOR is adaptable to patient performance, because each of its exercises has different levels of difficulty that the therapist can adjust to prevent boredom or frustration caused by their being too easy or too difficult, respectively; (c) GRADIOR includes images associated with the patient’s real life to enhance user entertainment; (d) GRADIOR issues a feedback message informing whether the patient has been right or wrong; (e) GRADIOR can be used on digital devices (touchscreen computers or tablets).

In recent decades, significant improvements in memory, perception and attention have been reported in dementia, as well as improvements in working memory and psychomotor learning in people with MCI through computer-based cognitive training [63]. Other studies support the efficacy of computer-based cognitive rehabilitation for people with cognitive impairment [52].

In a meta-analysis of 17 randomized clinical trials, Hill, Mowszowski [64] found statistically significant moderate effect sizes for verbal memory, non-verbal memory, working memory, attention and psychosocial functioning. Other systematic reviews and meta-analyzes also reported similar results, not only for people with cognitive impairment, but also for people with depression and anxiety [23].

Studies on the effectiveness of GRADIOR highlighted the improvement of auditory memory, verbal learning and concentration [65]. As well as, an improvement in the perception of functional capacities, increasing independence and social interaction [44].

Although GRADIOR yielded good results in different types of patients, more robust results are needed [65]. In order to study the effectiveness of the new "GRADIOR" version, from 2018 to the present, a randomized clinical trial framed within Initial Training Network (ITN) action, H2020-MSCA-ITN-2015, under grant agreement number 676265 is being carried out. Through this randomized clinical trial, we intend to evaluate the effectiveness of the GRADIOR rehabilitation program on cognitive functioning and social, emotional and functional aspects in people with MCI and mild dementia [66].

GRADIOR 4 is specific software for cognitive rehabilitation that uses the latest technology and takes into account the preferences of end-users and therapists. The involvement of end-users, therapists and other stakeholders [67] in the development of GRADIOR has led to the creation of a tool that is highly suitable and convenient for clinical settings, while also contributing towards the acceptability of GRADIOR by people with dementia.

We currently consider GRADIOR to be a highly usable tool in clinical practice for people with cognitive impairment caused by a broad range of pathologies. And because it allows distance therapy, it is accessible to people who are usually excluded from regular treatment because of the area they live in or mobility problems. GRADIOR’s flexibility makes the tool useful for many different pathologies.

Computer-based cognitive rehabilitation programs like GRADIOR can be provided as part of a comprehensive treatment, yielding good results in different modalities when combined the cognitive stimulation with physical training (LLM project^1^ involved GRADIOR 4) [68]. However, accurate software definition is essential, since this marks large differences. Consequently, to deem a computer program useful or not requires a thorough explanation of its features and the results of one software should not be deployed to all of them.

## Conclusions

In recent years, the number of older people with dementia in Europe has been growing, and it is estimated that approximately 30% of the population will suffer from some form of dementia by 2060 [1]. This has led to the development, improvement and implementation of different types of treatment, among which are non-pharmacological therapies involving psychosocial approaches and the use of new technologies such as GRADIOR.

GRADIOR is a computer-based cognitive rehabilitation program that allows the stimulation and training of cognitive functions in people with different neurological pathologies, including people with dementia. GRADIOR adjusts and responds to the characteristics and needs of people with dementia, producing greater usability of the software.

In this order of ideas, different studies on user experience, usability and effectiveness have been conducted. Users report a high degree of satisfaction with the use of the program, which turns out to be user-friendly and effective in helping to improve cognitive functions [36, 37, 40, 41, 43, 44]. Currently, new studies of user experience, usability and effectiveness for the new GRADIOR version continue to be carried out, pending the publication of their findings. These new studies will help to contrast and support the data already obtained in previous studies, providing more evidence to support the use of the program in a clinical or rural context with patients with cognitive impairment, which has been positive so far.

The version of GRADIOR in tablet is being developed with the aim that this program can be applied different contexts. For example, in the case of people with physical alterations, which could influence their displacement to specialized centers.

Finally, the development of a computer-based cognitive training program like GRADIOR contributes to the field of cognitive rehabilitation in people with cognitive impairment. This field characterized by pencil and paper stimulation has grown in recent years with the development of new programs that contribute to and help maintain cognitive performance in people with impaired cognitive functions. And therefore, to produce positive effects on the quality of life of the patient; for example, increasing their mood and even the social interaction with other people [43]. This type of treatment is contrary to pharmacological, the latter is usually the most used, but not always the most suitable for the rehabilitation of cognitive deficits.

## Supplementary information

**Additional file 1.** GRADIOR PROGRAM.

## Data Availability

Not applicable.

## Abbreviations

CAMCOG: Cambridge Cognition Examination
CDT: Clock Drawing Test
CIE-10: International Diagnosis of Diseases
GDS: Geriatric Depression Scale Yesavage
IADL: Lawton Instrumental Activities of Daily Living questionnaire
ICT: Information and Communications Technology
MCI: Mild cognitive impairment.
MMSE: Mini Mental State Examination
TMT: Trail Making Test

## Footnotes

1 Long Lasting Memories Project is partially funded under the ICT Policy Support Programme (ICT PSP) as part of the Competitiveness and Innovation Framework Programme by the European. Project number: 238904.
