# Supplementary material for: Computer-based cognitive rehabilitation program GRADIOR for mild dementia and mild cognitive impairment: new features
Source: BMC Med Inform Decis Mak. 2020 Oct 22;20:274. doi: 10.1186/s12911-020-01293-w (PMC7584078; doi:10.1186/s12911-020-01293-w)
Supplement: Supplementary file 1 — Additional file 1. GRADIOR PROGRAM. [file 12911_2020_1293_MOESM1_ESM.docx]

**Additional file 1: GRADIOR PROGRAM**

You can access these links and see some exercises associated with the GRADIOR program

Click on the following link. The password is alejandra

<https://owncloud.intras.es/public.php?service=files&t=94af607550ccbe124dd3f0805f9244f6>

Click on the following links and you can see exercises associated with the GRADIOR program.

<https://vimeo.com/132312470>

<https://vimeo.com/132312471>

<https://vimeo.com/132306589>

<https://vimeo.com/132312472>

<https://vimeo.com/132306584>

<https://vimeo.com/132306585>

<https://vimeo.com/132306587>
